# Metastatic Ovarian Clear Cell Carcinoma in the Context of *In Vitro Fertilization* Pregnancy

**DOI:** 10.1155/2020/2695058

**Published:** 2020-01-03

**Authors:** Arati Inamdar, Adriana Fulginiti, Shuk Fong Yiu, Abraham Loo, Brian Rogers, Robert Massaro, Robert A. Graebe, Thomas E. Hackett

**Affiliations:** ^1^Department of Pathology, RWJBarnabas Health, Monmouth Medical Center, Long Branch, NJ 07740, USA; ^2^Department of Obstetrics and Gynecology, Monmouth Medical Center, Long Branch, NJ 07740, USA; ^3^Department of Pathology and Laboratory Medicine, Rutgers-Robert Wood Johnson Medical School, New Brunswick, NJ 08901, USA

## Abstract

Adnexal masses are routinely encountered in the clinical practice. However, adnexal masses during pregnancy are incidental findings and usually resolve spontaneously or can be managed conservatively during pregnancy due to their benign nature. Ovarian malignancy is a rare event to occur during pregnancy. Only a few cases of ovarian clear cell carcinoma (OCCC), a subtype of epithelial ovarian cancers, have been reported in pregnancy and all of which have undergone cystectomy or pregnancy termination prior to the last trimester of pregnancy. We present a unique case of OCCC in a pregnant 38-year old female of Asian ethnicity with endometriosis and an *in vitro fertilization* (IVF) pregnancy. The OCCC, initially suspected to be of benign nature, was removed via emergency cesarean section during delivery in the late preterm period. The Positron Emission Tomography scan performed a few weeks after delivery confirmed metastatic lesions. Our case study not only emphasizes the need for definitive treatment option for endometriosis but also a close surveillance of all masses diagnosed during pregnancy, in particular with a background of other risk factors such as endometriosis and Asian ethnicity. In addition, our study advocates the need for the guidelines for management of such rare cases.

## 1. Introduction

Adnexal masses during pregnancy, although infrequent, are usually incidental findings. Masses presenting during early pregnancy often regress as the pregnancy progresses [1]. Masses persisting after the first trimester are generally excised to prevent torsion or rupture during the pregnancy and to exclude malignancy. A transvaginal ultrasound (TVUS) can often distinguish benign from malignant ovarian masses and thus guide in the management of pregnant patients with such masses [2]. The risk of ovarian malignancy is rare in pregnancy with a reported incidence of approximately 1 in 50,000 [3].

Epithelial ovarian carcinoma (EOC) constitutes about 90% of the ovarian malignancies and is considered as the most lethal gynecologic malignancy [4]. It consists of various histologic subtypes such as high-grade serous, clear cell, endometrioid, low-grade serous, mucinous and others [5]. Ovarian clear cell carcinoma (OCCC) is the second most common subtype after high-grade serous carcinoma (HGSC) and represents 5–10% of all EOCs in North America with relatively higher prevalence in East Asian region [5]. OCCC occurs in premenopausal women with a mean age of 50–55 years although some cases have also been reported in younger populations [5]. OCCC is often associated with endometriosis especially in 5–15% of the reproductive population and is proposed to be due to malignant transformation of endometrial focus [6]. The genetic profile of OCCC is characterized by *ARID1A (AT-rich interaction domain 1A)* and *PIK3CA (phosphatidylinositol-4,5-bisphosphate 3-kinase catalytic subunit alpha)* mutations, *MET (mesenchymal-epithelial transition)* amplification, and rare p53 mutation [7].

To our knowledge, the cases of ovarian malignancies so far reported during pregnancy have undergone cystectomy or termination of pregnancy prior to the last trimester of pregnancy [8, 9]. Our 38-year old patient possessed a triad of risk factors for aggressively growing mass during pregnancy, namely endometriosis, *in vitro fertilization*, and Asian ethnicity. The mass, pathologically characterized as OCCC, was removed at the time of cesarean section in the late-preterm period. The PET scan performed a few weeks after delivery demonstrated wide spread metastasis for which the patient has been undergoing multiple rounds of chemotherapy and radiation. Our unique case of metastatic OCCC removed during the third trimester of pregnancy highlights the importance of awareness regarding the signs and symptoms of adnexal mass and their close surveillance in female of reproductive age. In addition, it sheds light on the need for definitive treatment for endometriosis in the reproductive age and early management of all adnexal masses identified during pregnancy.

## 2. Case Report

**Figure 1:**
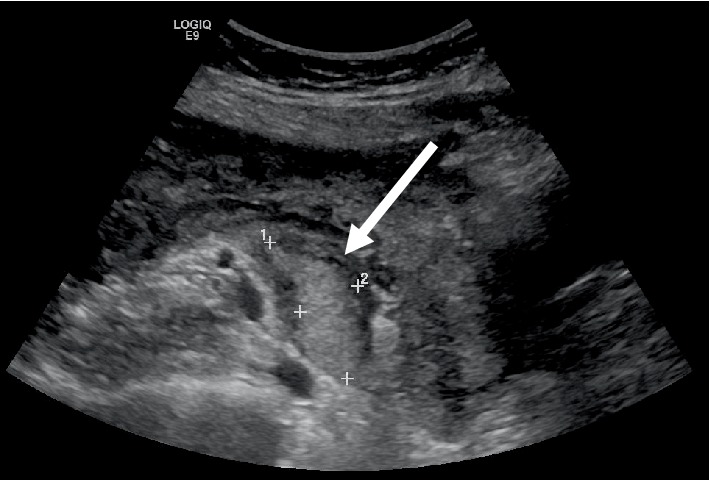
Ultrasonography of pelvis: the right ovary with an echogenic mass (arrow) measuring 4.2 × 2.1 × 3.8 cm is identified.

This case presents a 38-year-old Asian G2P1001 female who delivered by cesarean section (CS) at 32 weeks gestation for pre-eclampsia with severe features with uncontrollable blood pressures. Her pregnancy was further complicated by a known right ovarian mass, history of endometriosis which was managed conservatively, as well as infertility. Both first and second pregnancies were conceived via *in vitro fertilization* by frozen embryo transfer. Her first pregnancy was otherwise uncomplicated, however resulted in primary CS for nonreassuring fetal heart tracing. No pelvic pathology was noted during that CS. The patient was first diagnosed with a right ovarian mass during her second pregnancy while on IVF treatment. The patient declined the option of removing the mass due to possibility of damaging the nearby ovarian tissue during removal of the mass and thus worsening her infertility. The IVF treatment was successful. The transvaginal ultrasonography performed during the early pregnancy suspected the right ovarian mass as endometrioma or desmoid tumor (Figure 1).

The mass was kept under surveillance by performing periodic ultrasound imaging. Due to the change in mass characteristics and increase in size, the patient was referred to a Gynecologic Oncologist during second trimester. Considering the high risk pregnancy, recommendation was made to follow the mass with sequential ultrasound with the removal of the mass at the time of repeat CS as long as the mass did not change in size or configuration during the pregnancy and patient remained asymptomatic. Unfortunately, the patient developed pre-eclampsia with severe features at 32 weeks of gestation. Upon admission, she was given magnesium sulfate infusion for seizure prophylaxis and corticosteroids for fetal lung maturity. The Maternal-Fetal-Medicine specialists recommended delivery at thirty-four weeks of gestation unless there were new signs of maternal or fetal instability. However, three days after the admission, she underwent an emergency cesarean section due to recurrent severe blood pressure changes which were unresponsive to intravenous antihypertensive medications.

During cesarean section, extensive adhesions were noted at the level of adipose tissue, rectus muscle, and anterior surface of the uterus, which were densely adhered to each other and to the anterior abdominal wall. With limited visualization of the lower uterine segment, a classical vertical uterine incision was performed. The fetus was delivered without difficulty. The uterus was exteriorized and closed in layers. At this point, the right ovarian mass was visualized, the surface of which was friable and hemorrhagic. Two units of each packed red blood cells, fresh frozen plasma, and cryoprecipitate were administered. Antibiotics were re-dosed due to prolonged surgical time. With extensive lysis of adhesions and uterine packing, the surgeons successfully ligated the uterine pedicle and the infundibulopelvic ligament to remove the large ovarian mass.

**Figure 2:**
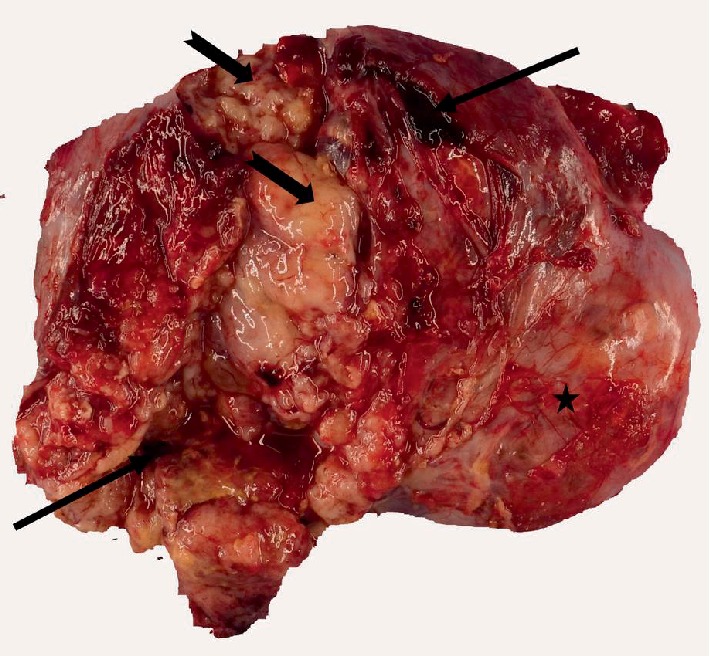
Gross photograph of the ovarian mass: the gross appearance of ovarian mass demonstrating the necrotic material (notched arrows) and hemorrhage (arrows) with smooth external surface (star).

The pathological examination of the resected specimen revealed an ovarian mass with attached intact fallopian tube, weighing 63 g and measuring 14.0 × 10.5 × 3.0 cm (Figure 2).

**Figure 3:**
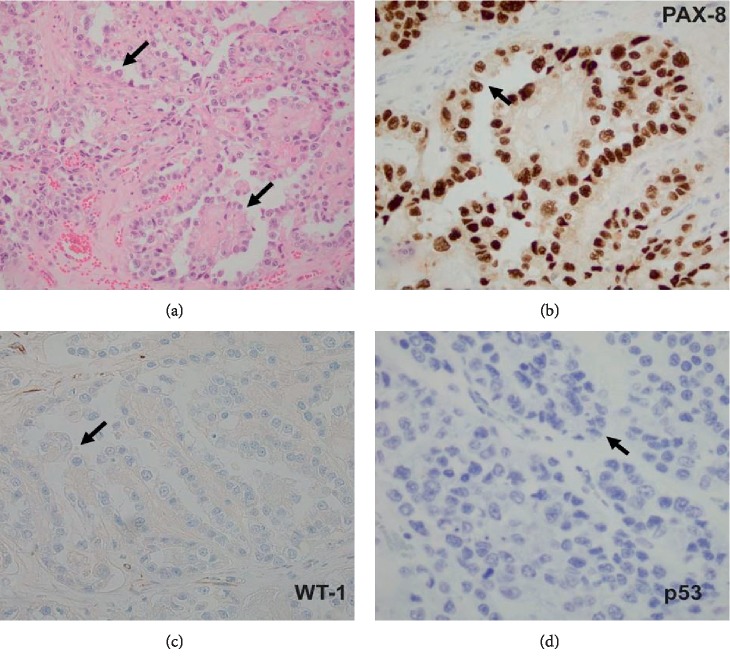
Ovarian mass showing features of ovarian clear cell carcinoma. (a) The hematoxylin and eosin stain demonstrating tubulocystic and papillary architecture along with focal areas of solid sheets of tumor cells consists of hobnail cells (arrows). (b) The immunohistochemical stains demonstrating positive reactivity (brown stain-arrow) for PAX-8. (c) and (d) The immunohistochemical stains demonstrating negative reactivity (lack of brown stain-arrows) for WT-1 and p53, respectively.

The external surface of ovarian mass was smooth, hemorrhagic but without any excrescences. The serial sectioning revealed multiple cystic lesions filled with clear to yellow fluid as well as necrotic material overall occupying 80% of the ovarian mass. Microscopically, the viable ovarian tissue demonstrated tubulocystic and papillary architecture along with focal areas of solid sheets of tumor cells displaying the clear cytoplasm. Multiple areas of hyperchromatic nuclei with conspicuous nuclei (hobnail cells) were evident (Figure 3(a)). The immunohistochemistry for Wilm's tumor-1 (WT-1) and wild type p53 were negative while positive for Pax-8 (Figures 3(b)–3(d)). The findings were indicative of high grade ovarian clear cell carcinoma without expression of wild type or mutant p53.

## 3. Discussion

Adnexal/ovarian masses during pregnancy are usually incidental findings. Ultrasound and Magnetic Resonance Imaging (MRI) are considered as safe diagnostic tools to distinguish between benign and malignant masses with high predictive value, especially when considering the tumor size, morphology and color doppler flow [9]. MRI-based assessment of ovarian masses is employed when ultrasound diagnosis is questionable, if the mass is suspiciously large, or when evaluation of possible extra-ovarian spread is required in light of possible malignant mass [10]. Only 3–5% are of ovarian masses occurring during pregnancy are of malignant nature [3]. All subtypes of epithelial ovarian cancer have been reported during pregnancy [8]. Although there is no defined guideline for the management of such ovarian masses occurring during pregnancy, there are the options of open surgery, laparoscopy, or “wait and watch”. The nature of the ovarian mass, its size, stage if malignant, gestational age and available surgical expertise must be considered when deciding on the route of management [11].

Among EOCs, OCCC is considered to be the most aggressive subtype and accounts for 5% of epithelial ovarian cancers. Their mean diameter is fifteen centimeters and can be solid, usually with yellow nodules and thick-walled unilocular cysts that often contain watery or mucinous fluid [12]. It is interesting to note that the incidence of OCCC in Caucasian women is 4.6%, however; it is more common in Asian and Japanese women with incidence rate as high as 11% and 25%, respectively; the reason for which is still unknown although role of a genetic factor is one possibility [12].

Furthermore, OCCC is known to be associated with endometriosis and atypical adenofibroma with a relative risk of 12.5% [13]. Endometriosis is a condition of ectopic endometrial tissue, often resulting in infertility. Although rare, 2.5% of ovarian endometriosis cases result in malignant transformation mostly due to K-ras associated mutation and often are diagnosed at a relatively early age [13, 14]. These patients often have a better prognosis than women with malignant ovarian cancer without endometriosis [15]. Interestingly, tubal ligation has been proven to be protective against development of clear cell carcinoma as the occluded tubes prevent retrograde disposition of endometriotic lesions [16].

Several small and large case-control studies have explored the relationship between fertility drug use during IVF treatment and risk of ovarian cancer [17, 18]. The IVF treatment involves use of fertility drugs to induce maturation of follicles and ovulation by elevating the gonadotropin levels [19]. Although the clinical data from some of the early studies demonstrate the increased risk for ovarian cancer, recent studies have failed to validate such association [18]. It is worth noting that few studies have suggested that the risk of ovarian cancer increases with the number of IVF cycles, especially with more than 12 cycles. The pathophysiology behind this can be explained by repeated damage and stimulation of the ovarian epithelial surface of ovary by IVF drugs [17, 20]. Overall, results are controversial and large well-designed studies are needed to further confirm the relationship between IVF drugs and ovarian cancer.

Of the reported benign and malignant ovarian masses occurring during pregnancy, all have been reported to be removed during the first or second trimester [8]. In one published study of a 37-year old woman with a history of infertility, endometriosis and two prior unsuccessful IVF treatments, OCCC was diagnosed at six week of gestational age with the mass measuring 6.0 × 4.0 cm. The exploratory laparotomy and cystectomy performed at 14 weeks confirmed the mass to be OCCC of stage Ic, arising in the endometriosis. The pregnancy was continued with a delivery of newborn baby at 34 weeks by cesarean section accompanied by hysterectomy, bilateral salpingo-oophorectomy and omentectomy without any recurrence of the OCCC in the resected specimen [9].

CA 125 is usually elevated in ovarian tumors. Unfortunately, there is a limited reliability of CA 125 as a cancer marker in a pregnant patient with endometriosis since it is normally elevated during pregnancy [21]. In terms of prognosis, OCCC when presented at the early stage (stage I and II) is treated with a platinum-agent and taxane [22]. However, late stage OCCCs are especially notorious for being chemo-resistant to platinum based chemotherapy mostly due to presence of genetic alterations in *PIK3CA*, *ARID1A*, and *MET* genes. The most common therapeutic approach for metastatic OCCC is debulking, chemotherapy and radiation therapy [23]. In our patient, the PET scan performed few weeks after delivery revealed metastases in the long bones and vertebral column. She is currently undergoing multiple rounds of chemotherapy along with radiation at other institution, further details of which are not available.

## 4. Conclusion

Our case emphasizes the importance of close imaging surveillance for all masses diagnosed during the pregnancy in particular with a background of other risk factors such as endometriosis, East Asian ethnicity and IVF. The gravid females who undergo fertility treatment with a pre-existing ovarian mass and underlying endometriosis require close monitoring and early surgical management since they are at risk for rapid growth and malignant transformation.
